# Peak Cardiac Mechanical and Haemodynamic Responses During Incremental Exercise Are Temporally Associated With Ventilatory Thresholds: Novel Insight Into the Coordination of Integrative Physiological Responses to Exercise

**DOI:** 10.1111/apha.70331

**Published:** 2026-11-02

**Authors:** Fabian Spahiu, Moritz Lampkemeyer, Michelle Ottlik, Max Hagemann, Lars C. Helbig, Eric J. Stöhr

**Affiliations:** ^1^ COR‐HELIX, Institute of Sport Science Leibniz University Hannover Hannover Germany; ^2^ Rehabilitation Centre Klinikum Kassel Kassel Germany

## Abstract

**Background:**

Maximal aerobic capacity (VO_2_peak) is traditionally attributed to limitations in oxygen transport and utilization. However, it remains unclear whether peak cardiac mechanical and haemodynamic responses occur in temporal association to ventilatory thresholds during incremental exercise, and whether these relationships vary with cardiorespiratory fitness.

**Methods:**

Eighteen healthy, recreationally active adults (26 ± 3.18 years VO_2_peak 41.87 ± 4.76 mL·kg·min^−1^) completed two incremental supine cycling tests to exhaustion, with breath‐by‐breath gas exchange analysis, exercise echocardiography, and continuous blood pressure monitoring. Plateau timing (PT) was defined as the relative heart rate (%HRmax) at which each cardiovascular variable reached its peak or plateau response. Associations between cardiovascular PTs, VO_2_peak, and the first (VT1) and second ventilatory thresholds (VT2) were examined using correlation analysis. Exploratory k‐means clustering was used to descriptively visualize plateau‐timing distributions.

**Results:**

VT1 significantly correlated with PT–ejection fraction (EF) and PT–vascular conductance, whereas VT2 correlated with deformation‐based parameters including PT–apical circumferential strain, PT–apical rotation, and iCarT (*p* < 0.05). VO_2_peak was positively associated with PT–twist and PT–apical rotation (*p* < 0.05), and negatively associated with PT–EF (*p* < 0.05). Exploratory clustering yielded two descriptive clusters which differed in VO_2_peak (*p* < 0.05) and were characterized by differences in volumetric and rotational plateau‐timing distributions.

**Conclusions:**

During exercise, different cardiac parameters are temporally associated with ventilatory thresholds 1 and 2. Cardiorespiratory fitness may influence interindividual differences in the temporal organization of response patterns during incremental exercise.

## Practitioner Points

- Selected cardiac mechanical and haemodynamic responses during incremental exercise were temporally associated with ventilatory thresholds.
- Aerobic capacity is associated with the timing of these cardiac responses, with higher VO_2_peak linked to a distinct pattern of mechanical and volumetric adaptation during incremental exercise.
- These findings support an integrated interpretation of cardiopulmonary exercise testing, in which metabolic thresholds may reflect coordinated systemic and cardiac adaptations rather than metabolic events alone.

## 1 Introduction

During incremental exercise to volitional fatigue, the integrative physiological responses are characterized by non‐linear dynamics. In this context, whole‐body oxygen uptake (VO_2_) increases approximately linearly up to high exercise efforts but has been reported to eventually reach a plateau prior to maximal exertion, thought to reflect the upper limit of aerobic energy turnover [1]. VO_2_max has traditionally been interpreted as evidence of a central limitation arising from the inability to further increase oxygen delivery to the working musculature [2]. Accordingly, maximal aerobic capacity has largely been conceptualized within a central limitation framework, whereby whole‐body oxygen uptake is primarily constrained by the convective transport of oxygen‐rich blood to the working musculature, while peripheral oxidative capacity and diffusive limitations are considered to play a subordinate role in healthy humans [3, 4, 5]. Strong support for this view has been provided by isolated muscle exercise models, demonstrating that when active muscle mass is restricted, skeletal muscle is capable of sustaining substantially higher blood flows and oxygen uptake than observed during whole‐body exercise, indicating that peripheral perfusion and mitochondrial capacity are not inherently limiting under physiological conditions [6, 7].

Within this framework, the contribution of stroke volume (SV) and its regulation during exercise reflect the integrated interaction of ventricular filling (preload), myocardial contractility, afterload, and heart rate [4, 8]. Consistent with the classical concepts proposed by Krogh and Lindhard as well as Guyton, the augmentation of SV during exercise is fundamentally dependent on ventricular filling, with the Frank–Starling mechanism contributing substantially to increases in SV, particularly during the transition from rest to moderate exercise intensities [9, 10, 11]. However, despite the recognized importance of preload‐mediated mechanisms, accumulating evidence suggests that different cardiovascular domains do not necessarily exhibit synchronous response patterns during incremental exercise. Volumetric responses, particularly end‐diastolic volume (EDV), have been shown to plateau relatively early during exercise, indicating a lower contribution of Frank–Starling‐mediated augmentation of SV during higher exercise intensities [2, 12, 13]. In contrast, indices of myocardial deformation derived from two‐dimensional speckle‐tracking echocardiography, including global longitudinal strain, circumferential strain, left ventricular twist and untwisting rate, may continue to increase beyond the point at which volumetric responses have plateaued, with left ventricular twist in particular frequently reaching its peak at higher exercise intensities [12, 13]. Together, these observations suggest that volumetric, haemodynamic, and myocardial mechanical responses may follow distinct temporal trajectories during incremental exercise rather than converging at a single cardiovascular ceiling. However, evidence supporting these interactions have largely been based on group‐level analyses, thereby obscuring potentially meaningful interindividual variability in the temporal organization of cardiovascular responses.

To address this limitation, we recently introduced the concept of the individual cardiac contraction threshold (iCarT), defined as the relative heart rate at which left ventricular (LV) deformation reaches its maximum during step‐wise incremental exercise [12]. Using this approach, we observed substantial interindividual variability in the timing of different indices of LV deformation, with LV twist most frequently determining iCarT and typically plateauing later than other deformation indices. Importantly, in previous investigations, iCarT did not temporally coincide with the individual SV plateau. Rather, peak myocardial mechanical responses generally occurred at higher relative exercise intensities than peak SV responses, suggesting that myocardial mechanical and volumetric responses may follow distinct temporal trajectories during incremental exercise [12].

This previous observation raises a broader physiological question regarding the temporal organization of cardiovascular responses during exercise. Specifically, it remains unclear whether the timing of peak cardiovascular responses is linked to established integrative transitions in whole‐body physiology or whether different cardiovascular domains operate relatively independently. In this context, ventilatory thresholds represent well‐established physiological landmarks characterized by coordinated changes in pulmonary gas exchange, substrate utilization, autonomic regulation, and acid–base balance [14, 15]. Although ventilatory thresholds have been associated with global cardiovascular markers such as heart rate variability (HRV) and heart rate deflection points [16, 17], it remains unknown whether they are temporally associated with peak responses across distinct cardiovascular domains. In particular, it is unclear whether peak volumetric responses of the left ventricle, reflected by EDV, end‐systolic volume (ESV), and SV, peak myocardial mechanical responses, and peak peripheral haemodynamic responses, including vascular conductance (VC) and systemic vascular resistance (SVR), occur in temporal proximity to the first and second ventilatory thresholds (VT1 and VT2), or whether these physiological events follow independent temporal trajectories. Clarifying these relationships will provide new insight into the integrative organization of exercise physiology and improve our understanding of how cardiovascular and metabolic responses interact during progressive exercise. Given that ventilatory thresholds are widely used to guide exercise prescription and interpret physiological performance, identifying whether specific cardiovascular domains exhibit distinct temporal associations with these thresholds may also refine the physiological interpretation of threshold‐based training concepts and exercise testing. Furthermore, a more differentiated characterization of peak cardiovascular responses during exercise may ultimately contribute to a more nuanced assessment of cardiovascular adaptation and reserve in both athletic and clinical settings.

Accordingly, the primary aim of the present study was to determine whether the timing of peak cardiovascular responses across volumetric, haemodynamic, and myocardial mechanical domains exhibits distinct temporal associations with VT1 and VT2 during incremental exercise, or whether these responses converge at a common physiological ceiling. As a secondary, exploratory aim, we examined whether cardiorespiratory fitness may further influence the temporal organization of these peak responses. We hypothesized that peak cardiovascular responses would not occur simultaneously across physiological domains but instead follow a parameter‐specific temporal sequence. Specifically, we expected VT1 to be more closely associated with earlier peak or plateau attainment in volumetric and peripheral haemodynamic indices, whereas VT2 would be more closely associated with the timing of peak or plateau attainment in myocardial mechanical indices. Furthermore, we hypothesized that individuals with greater cardiorespiratory fitness would exhibit a delayed attainment of peak or plateau responses, particularly in myocardial mechanical domains, reflecting a more sustained cardiovascular reserve during incremental exercise.

## 2 Methods

### 2.1 Ethical Approval, Study Design and Population

The study was approved by the local ethics committee (Leibniz University Hannover, reference number EV LUH 14/2024), and written informed consent was obtained from all participants prior to data collection. A total of 21 healthy recreationally active individuals were recruited and tested. The present study is part of a larger exploratory research project from which additional data have already been published [12]. The datasets of three participants were excluded from the overall analysis due to insufficient echocardiographic image quality and/or technical issues with continuous blood pressure monitoring during Visit 2. As a result, the final analysis included data from 18 participants with a mean age of 26 ± 3.18 years, a mean height of 180.1 ± 8.1 cm, body mass of 77.8 ± 11.1 kg, a body mass index (BMI) of 24.01 ± 2.36 kg/m and a peak oxygen uptake (VO_2_peak) during supine, tilted incremental exercise testing of 41.87 ± 4.76 mL kg min^−1^. Of the 18 participants, five (28%) were women. The mean resting brachial systolic blood pressure (SBP) was 125 ± 11 mmHg, whereas the mean resting diastolic blood pressure (DBP) was 79 ± 6 mmHg. The average pulse wave velocity at rest was 5.16 ± 0.49 m/s. Baseline characteristics of the study population are presented in Table 1. Because data availability differed across physiological domains, the effective sample size varied by outcome and is reported separately for each analysis, with participant‐level data attrition and reasons for missing measurements detailed in Figure S1. This study was designed as an exploratory physiological investigation of individual cardiovascular plateau behavior during incremental exercise.

**TABLE 1:** Participant characteristics and peak exercise values (*N* = 18).

Parameter	Mean ± SD
Age (years)	26 ± 3.18
HR (bpm)	70 ± 11
SBP (mmHg)	125 ± 11.26
DBP (mmHg)	79 ± 6.12
PWV (m/s)	5.16 ± 0.49
Height (cm)	180.06 ± 8.09
Body mass (kg)	77.83 ± 11.09
BMI (kg/m^2^)	24.01 ± 2.36
SV (mL)	72.58 ± 15.90
EF (%)	55 ± 4.2
W_peak_ (watts)	226 ± 39.16
HR_max_ (bpm)	179 ± 12.70
VO_2_peak (mL kg min^−1^)	41.87 ± 4.76

*Note:* Values are presented as mean ± SD.

Abbreviations: BMI, body mass index; DBP, diastolic blood pressure; EF, ejection fraction; HR, heart rate; HR_max_, maximal heart rate; PWV, pulse wave velocity; SBP, systolic blood pressure; SV, stroke volume; VO_2_peak, peak oxygen uptake; W_peak_, maximal workload.

All participants underwent two consecutive laboratory visits as part of a standardized exercise testing protocol at the COR‐HELIX laboratory of the Leibniz University in Hannover, Germany. Data acquisition took place between July 2024 and March 2025. Participants were recruited from lectures at the Institute of Sports Science of the Leibniz University and via social media platforms. Inclusion criteria were an age over 18 years and a physically active lifestyle, defined as engaging in any form of exercise on more than 2 days per week. Exclusion criteria included any kind of known cardiac abnormalities, medication for acute or chronic disease (except male or female contraception) and poor image quality during baseline echocardiography. Visit 1 served as a screening to assess eligibility for study participation and to determine the individual peak work capacity (W_peak_). During Visit 2, all individuals underwent an individualized step‐wise incremental exercise protocol in order to determine ventilatory thresholds, VO_2_peak, cardiovascular plateau timing (PT) including the previously described individual cardiac contraction threshold [12].

### 2.2 Exercise Protocol and Study Design

Before exercise initiation during Visit 1, baseline characteristics including height, body mass, blood pressure, and pulse wave velocity were collected from all participants. Following a 10‐min seated rest period, brachial blood pressure and pulse wave velocity (PWV) were measured using an oscillometric device (Mobil‐O‐Graph, IEM, Aachen, Germany) in the seated position with the cuff placed at heart level. Three consecutive measurements were obtained at 2‐min intervals, and the mean values of blood pressure and PWV were used for subsequent analyses. Height and body mass were assessed using a stadiometer and scales (Soehnle, Nassau an der Lahn, Germany). Subsequently, participants were placed in a supine position on a recumbent bicycle ergometer (ergoselect 1200, ergoline, Bitz, Germany) to individually adjust the bike settings prior to the exercise test. During a 5‐min resting phase, suitability for exercise echocardiography was determined by checking the echocardiographic imaging windows and the quality of the images. If image quality was insufficient, the procedure was discontinued prior to the stress test, and the participant was excluded from the study. If the participant was classified as eligible, the stress test was initiated. The protocol at Visit 1 began at 50 W with 40 W increases every 3 min until volitional exhaustion was reached. The obtained W_peak_ was then used to individualize the subsequent testing protocol for Visit 2.

At Visit 2, participants performed individualized exercise starting at 40% of the W_peak_ identified during Visit 1, with the workload subsequently increasing by 10% of W_peak_ every 3min. The test continued until volitional fatigue was reached. This individualized stage design was chosen to standardize relative exercise intensity across participants while providing sufficient time within each stage for stable echocardiographic, gas‐exchange, and haemodynamic acquisition. Cardiac ultrasound imaging was performed during the final 90 s of each stage. Respiratory gases were continuously measured via breath‐by‐breath analysis (Quark CPET, COSMED, Schweinfurt, Germany). Continuous beat‐to‐beat blood pressure was recorded using photoplethysmography (FinometerPRO, FMS, Enschede, Netherlands). Throughout testing, a dedicated investigator continuously monitored the Finometer signal, provided real‐time feedback to participants to minimize movement, and intervened when motion artifacts occurred to ensure stable signal quality and valid blood pressure recordings. SVR was calculated based upon established approaches according to the standard formula (MAP × 80)/Q with MAP denoting mean arterial pressure (mmHg) and Q denoting cardiac output (L min^−1^) [18]. VC was calculated as Q divided by mean arterial pressure (VC = Q/MAP) [19]. Due to intermittent signal loss and increased susceptibility to motion artifacts at higher heart rates, reliable blood pressure data across the full exercise protocol could only be obtained in a subset of participants; therefore, SVR and VC analyses were restricted to 12 individuals.

Participants exercised in a supine position and were tilted to the left during the 90 s of data acquisition to enable recording of the targeted ultrasound sequences. Left‐lateral positioning during image acquisition was standardized to 45° using the tilt mechanism of the ergometer. Tilting was restricted to the acquisition window to optimize acoustic windows while limiting prolonged posture‐related alterations in physiological responses and cycling mechanics. Importantly, echocardiographic outcomes were acquired consistently under the same acquisition posture at each stage, thereby standardizing any tilt‐related influence on the imaging measures across intensities. A consistent cadence of 60 to 70 rpm was maintained throughout the test. To control for physiological exertion and ensure a high level of effort, a respiratory exchange ratio (RER) ≥ 1.10 was used as a criterion for maximal exertion and was reached by all participants in Visit 1% and 70% of participants in Visit 2.

### 2.3 Echocardiographic Image Acquisition

For the quantification of two‐dimensional speckle tracking data, ultrasound images were acquired using a commercially available ultrasound system (Vivid E95, GE Medical, Horten, Norway). All data were collected by a trained cardiac sonographer using a multifrequency transducer (M5Sc D XDclear matrix single crystal phased array, GE Medical Systems Israel Ltd). Data acquisition followed the previously described procedures [20].

For the purpose of this study, the apical four chamber view, the parasternal short axis view at the left ventricular (LV) base and the parasternal short axis view at the apex of the LV were obtained at rest and during the final 90 s of each exercise stage. The LV‐base was defined as the short axis view showing the proximal aspects of the mitral valve leaflets during early diastole. The LV‐apex was obtained by aligning the transducer along the long axis of the LV, identifying the optimal 4‐chamber position and then moving caudally as little as possible, in a short‐axis angle, aiming to visualize the true apex while ensuring a circular lumen throughout the cardiac cycle and avoiding lumen obliteration at end systole. Each of the abovementioned images was acquired by using the highest frame rate possible in order to ensure optimal tracking quality and accuracy [21]. The frame rates for the different imaging windows were as follows: the apical four‐chamber view was acquired at 141.53 ± 11.88 frames per second, the parasternal short‐axis view at the basal level at 167.14 ± 24.27 frames per second, and the parasternal short‐axis view at the apical level at 233.00 ± 21.64 frames per second.

### 2.4 Echocardiographic Data Analysis

Echocardiographic image sequences were analyzed offline using EchoPAC software (EchoPAC version 204, GE, Trondheim, Norway) for initial data extraction by following the established guidelines [22]. In order to further improve the precision of the absolute left ventricular rotational and circumferential mechanics, the raw speckle‐tracking output obtained from the manufacturer software was further processed by interpolating data to 600 points with equidistant time intervals during systole and diastole, a method previously recommended by using a custom software tool (2D Strain Analysis Tool, version 1.0beta14, Stuttgart, Germany) [20, 23]. Basal rotation and rotation velocity curves (measured in degrees per second) were subtracted from the apical curves, respectively, to calculate peak systolic LV twist.

LS was determined from apical 4‐chamber images using the Automated Function Imaging (AFI) feature available in EchoPAC software (version 204, GE Ultrasound). AFI applies artificial intelligence for fully automated tracking of myocardial speckles, with the option to manually adjust regions of interest to ensure accurate tracking. In addition, AFI enables the simultaneous calculation of EDV, ESV, EF, SV, and Q based on endocardial border tracking within the same apical four‐chamber recordings.

Owing to inadequate apical four‐chamber images during at least one exercise stage, longitudinal strain and cardiac volumetric measures could not be obtained in 2 of 18 participants. Conversely, in another four participants, rotational and circumferential data could not be obtained. As a result, longitudinal strain and volumetric outcomes could be analyzed in 16 participants, and rotational/circumferential data were likewise available in 14 participants.

### 2.5 Determination of Plateaus in Cardiac Volumes, Deformation, and Haemodynamics

For each participant, curve‐fitting analyses were performed to characterize the relationship between relative exercise intensity, expressed as percentage of maximal heart rate achieved during Visit 2 (%HR_max_), and myocardial deformation, volumetric, and haemodynamic parameters, following the same analytical approach as applied in previous work from our group [12].

Polynomial regression models (quadratic or cubic) were applied to identify the relative heart rate at which each parameter reached its maximum or plateaued before either stabilizing, declining, or continuing to increase linearly across all exercise intensities. Model selection was based on corrected *R* ^2^ values and visual inspection of curve behavior. For quadratic models, maxima were determined using the vertex formula (*x* = −*b*/2*a*). For cubic models, the maximum was identified by solving the first derivative and determining the global maximum within the tested intensity range.

The individual cardiac contraction threshold (iCarT) was defined as the relative heart rate at which the last myocardial deformation parameter (longitudinal strain, circumferential strain, or LV twist) reached its maximum, reflecting the point at which myocardial contractile function no longer increased with rising exercise intensity. If no maximum or plateau could be identified and the response remained linear, iCarT was assigned a value of 1.0 (100% HRmax). In cases of a continuous negative association, a value of −1.0 was assigned. For participants in whom not all myocardial deformation parameters were available, iCarT was determined based on the deformation parameters that could be reliably quantified, using the same decision criteria as outlined above.

Importantly, the identical analytical framework was applied to volumetric indices (SV, Q, ESV, EDV, EF), blood pressure (SBP, DBP, MAP), and derived haemodynamic indices (VC and SVR). Plateau timing (PT) was defined as the relative heart rate at which a given parameter reached its maximal or stable plateau response during incremental exercise and is reported as PT‐Parameter (e.g., PT‐SV for stroke volume plateau timing).

To assess the sensitivity of the polynomial curve‐fitting approach to observations obtained at the highest exercise intensity, an additional leave‐final‐stage‐out sensitivity analysis was performed. For each participant and parameter, the final available exercise‐stage observation was removed and the complete curve‐fitting and plateau‐detection procedure was repeated using the same model‐selection and threshold‐detection criteria as in the primary analysis. Sensitivity‐derived plateau timings were compared with the original estimates by calculating the signed difference, absolute difference, median absolute difference with interquartile range, and range of individual differences. iCarT was subsequently recalculated from the sensitivity‐derived deformation plateau timings using the same definition as in the primary analysis. Finally, associations of the sensitivity‐derived plateau timings and iCarT with the ventilatory thresholds were reassessed using Spearman's rank correlations. This sensitivity analysis was performed as a secondary analysis to evaluate the methodological stability of plateau determination and was not part of the primary analytical framework. Detailed participant‐ and parameter‐level results are provided in the Supporting Information (Tables S1–S3).

### 2.6 Detection of Ventilatory Thresholds

Ventilatory thresholds were determined using breath‐by‐breath gas‐exchange data acquired with the COSMED Quark CPET system and analyzed in Omnia software (Version 1.6). Ventilatory threshold identification was performed visually according to established methodological criteria [24].

The first ventilatory threshold (VT1) was identified using the V‐slope method, defined as the intersection point of a two‐line regression of carbon dioxide output (VCO_2_) versus oxygen uptake (VO_2_), where the slope of the relationship increases from < 1 to ≥ 1. VT1 identification was supported by the presence of a nadir or first systematic increase in the ventilatory equivalent for oxygen (VE/VO_2_) without a concomitant increase in the ventilatory equivalent for carbon dioxide (VE/VCO_2_), as well as by a nadir or initial rise in end‐tidal oxygen pressure (PetO_2_) while end‐tidal carbon dioxide pressure (PetCO_2_) remained stable or continued to increase.

The second ventilatory threshold (VT2) was determined by visual inspection of ventilatory equivalents and end‐tidal gas behavior, in accordance with established criteria for the respiratory compensation point. Ultimately, VT2 was defined as the point at which a systematic increase or inflection in ventilation relative to carbon dioxide output occurred, reflected by a nadir or nonlinear increase in VE/VCO_2_, accompanied by a deflection or decline in PetCO_2_. Ventilatory thresholds were initially determined by one investigator using established criteria. All threshold determinations were subsequently reviewed by a second investigator and adjusted where necessary following consensus evaluation. Following threshold identification, the heart rate at the precise time point of each threshold was extracted. To minimize breath‐by‐breath variability, heart rate was averaged over a 15‐s window centered around the threshold time point. These averaged values were expressed as %HR_max_ using the highest heart rate obtained during Visit 2 as the reference.

VO_2_peak was determined as the highest 15‐s averaged VO_2_ value obtained during the incremental exercise test. This approach reduces transient variability and reflects the participant's highest sustained oxygen uptake during the final exercise stage.

### 2.7 Statistical Analysis

All statistical analyses were conducted using GraphPad Prism (version 10.6.0) and IBM SPSS (version 30.0). Data are presented as means ± SD. Non‐linear regression models were applied to characterize the shape and behavior of each cardiac deformation parameter in relation to relative heart rate.

The primary analyses examined associations between ventilatory‐threshold timing (VT1 and VT2, expressed as %HRmax) and cardiovascular plateau timing (PT). Secondary exploratory analyses examined associations of VO_2_peak with PT and with resting and peak cardiovascular variables.

At individual level, participant‐specific non‐linear regression models were fitted to each individual's data to capture individual volumetric, haemodynamic and cardiac contraction limitations.

At the group level, mean values for volumetric, haemodynamic, and cardiac contraction limitations were calculated, and the corresponding standard deviations were reported. Significance was set at an alpha level of 0.05. Depending on the distribution of the respective variables, as assessed using the Shapiro–Wilk test for normality, either Spearman's rank or Pearson's correlation coefficients were applied. Given the exploratory nature of this study, no formal correction for multiple testing was applied. Instead, exact *p*‐values and effect sizes are reported to facilitate interpretation and hypothesis generation.

To descriptively explore the distribution of cardiovascular plateau‐timing patterns within the present sample, an exploratory k‐means clustering analysis was performed using domain‐level features derived from individual plateau‐timing values. Variables were grouped a priori into volumetric (ESV, EDV, SV, EF), deformation (LS, basal circumferential strain, apical circumferential strain, basal rotation, apical rotation, twist, untwisting), and haemodynamic (Q, systolic and diastolic blood pressure, MAP, VC, SVR) domains. Ejection fraction (EF) was assigned to the volumetric domain, as it represents a composite index derived from ventricular volumes (EDV and ESV) and therefore most closely reflects volumetric function compared to the other domains. Cluster characteristics were summarized descriptively by domain‐level timing variables, parameter means, ventilatory thresholds, iCarT, and VO_2_peak. As VO_2_peak was not used as a clustering variable, differences in VO_2_peak between the two exploratory plateau‐timing profiles were assessed descriptively and using the Mann–Whitney *U* test.

## 3 Results

### 3.1 Associations Between Ventilatory Thresholds and Cardiovascular Plateau Timing

Relative HR at VT1 (VT1‐%HR_max_) was significantly associated with PT‐EF (*ρ* = 0.55, *p* = 0.035), and PT‐VC (*r* = 0.62, *p* = 0.030) (illustrated in Figure 1). VT1‐%HR_max_ was positively associated with PT‐SVR without reaching statistical significance (*ρ* = 0.53, *p* = 0.077). No significant associations between VT1‐%HR_max_ and plateau timing of other volumetric, haemodynamic, or deformation parameters (see Table 2) were identified.

**FIGURE 1:**
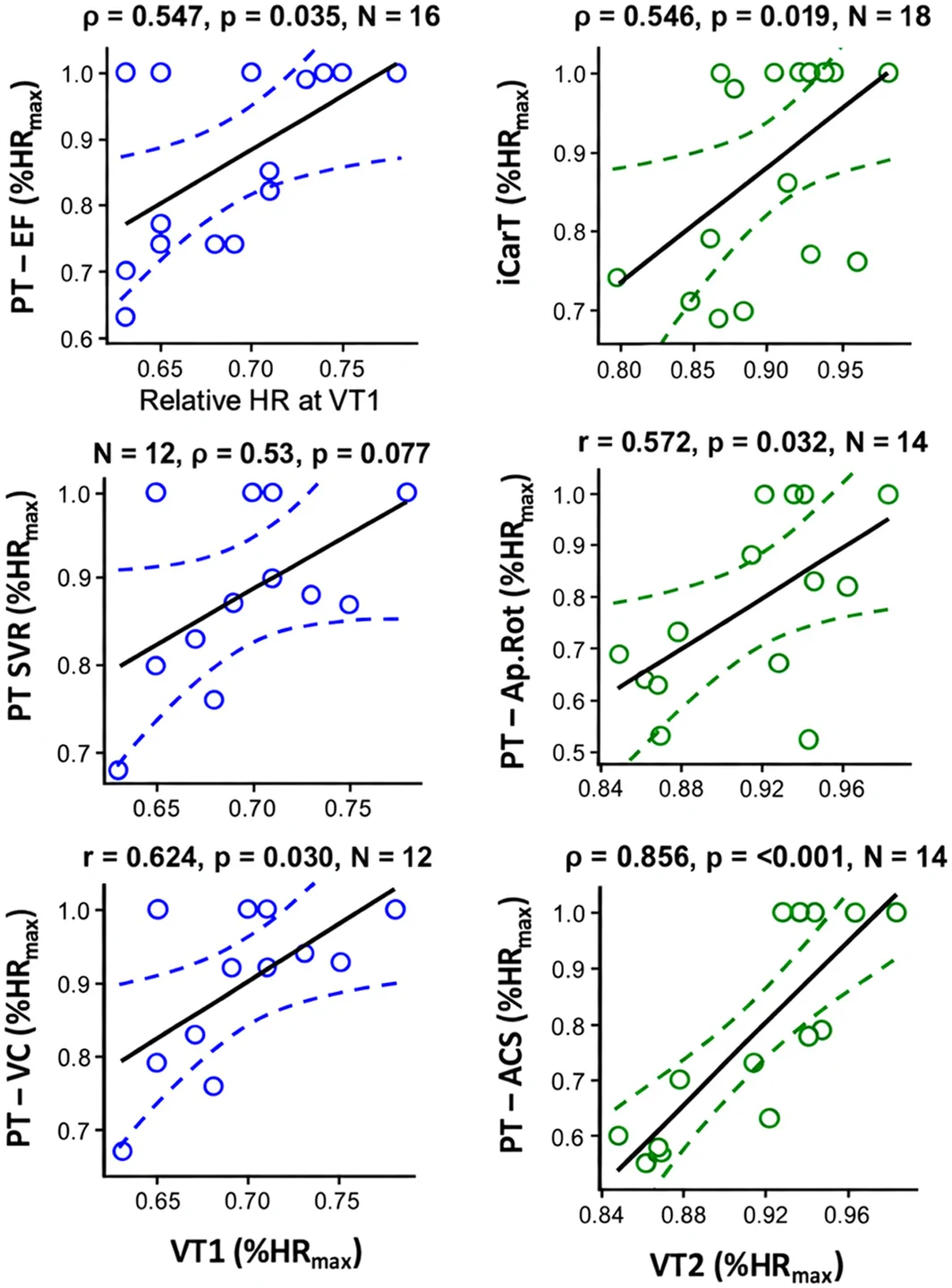
Associations between ventilatory thresholds and selected cardiovascular plateau timing. Scatterplots show associations between relative HR at ventilatory thresholds and relative HR at plateau formation of selected cardiac deformation and peripheral haemodynamic parameters. VT1 associations are shown in blue and VT2 in green. Left panels display VT1‐related associations; right panels display VT2‐related associations. Solid lines represent linear regression fits with 95% confidence intervals (dashed). Pearson's *r* or Spearman's *ρ* (according to distribution), corresponding *p*‐values, and sample sizes are indicated within each panel.

**TABLE 2:** Correlation between ventilatory threshold timing and the time point of cardiovascular plateaus.

	Correlation with VT1		Correlation with VT2	
	*r*/*ρ*	*p*	*r*/*ρ*	*p*
*Volumetrics, global cardiac function and LS (n = 16)*				
PT‐EDV	*ρ* = −0.07	0.802	*ρ* = −0.35	0.189
PT‐ESV	*ρ* = 0.02	0.956	*ρ* = 0.05	0.849
PT‐EF	*ρ* = 0.55	0.035*	*ρ* = −0.30	0.284
PT‐SV	*ρ* = −0.07	0.802	*ρ* = −0.35	0.189
PT‐Q	*ρ* = 0.22	0.414	*ρ* = 0.03	0.904
PT‐LS	*r* = 0.11	0.683	*r* = −0.16	0.568
*Myocardial deformation mechanics (n = 14) and iCarT (n = 18)*				
PT‐twist	*r* = 0.22	0.481	*ρ* = 0.48	0.085
PT‐Untwisting	*ρ* = −0.30	0.277	*ρ* = 0.50	0.071
PT‐ACS	*r* = 0.32	0.241	*ρ* = 0.86	< 0.001**
PT‐Ap.Rot	*ρ* = −0.14	0.611	*r* = 0.57	0.032*
iCarT	*ρ* = 0.13	0.586	*ρ* = 0.55	0.019*
*Blood pressure (n = 14)*				
PT‐SBP	*ρ* = 0.20	0.453	*ρ* = 0.24	0.399
PT‐DBP	*ρ* = −0.39	0.148	*ρ* = −0.02	0.956
PT‐MAP	*r* = −0.30	0.275	*r* = 0.42	0.137
*Peripheral haemodynamics (n = 12) and aerobic capacity (n = 18) *				
PT‐VC	*r* = 0.62	0.030*	*r* = 0.03	0.924
PT‐SVR	*ρ* = 0.53	0.077	*ρ* = −0.11	0.732
VO_2_peak	*ρ* = −0.12	0.646	*ρ* = 0.43	0.078

*Note:* PT denotes the relative heart rate at which the respective cardiovascular parameter reached its plateau during incremental exercise. Correlations are reported as Pearson's correlation coefficient (*r*) or Spearman's rank correlation coefficient (*ρ*) according to bivariate normality (Shapiro–Wilk test). Sample size (*n*) varied across parameters due to differences in data availability.

* *p* < 0.05 indicates a statistically significant association.

** *p* < 0.001 indicates a highly statistically significant association.

Relative HR at VT2 (VT2‐%HRmax) was significantly associated with PT‐ACS (*ρ* = 0.86, *p* < 0.001) and PT‐Ap.Rot (*r* = 0.57, *p* = 0.032), as well as iCarT (illustrated in Figure 1). VT2‐%HRmax was positively associated with PT‐twist (*ρ* = 0.48, *p* = 0.085) without reaching statistical significance. No significant associations between VT2‐%HRmax and plateau timing of left‐ventricular volumetric, haemodynamic, or other deformation parameters (see Table 2) could be identified.

### 3.2 Association Between VO_2_peak and the Timing of Cardiovascular Plateaus

VO_2_peak was significantly associated with PT‐twist (*ρ* = 0.63, *p* = 0.015) and PT‐Ap.Rot (*r* = 0.63, *p* = 0.015) (illustrated in Figure 2). VO_2_peak was positively associated with iCarT (*ρ* = 0.40, *p* = 0.104) without reaching statistical significance.

**FIGURE 2:**
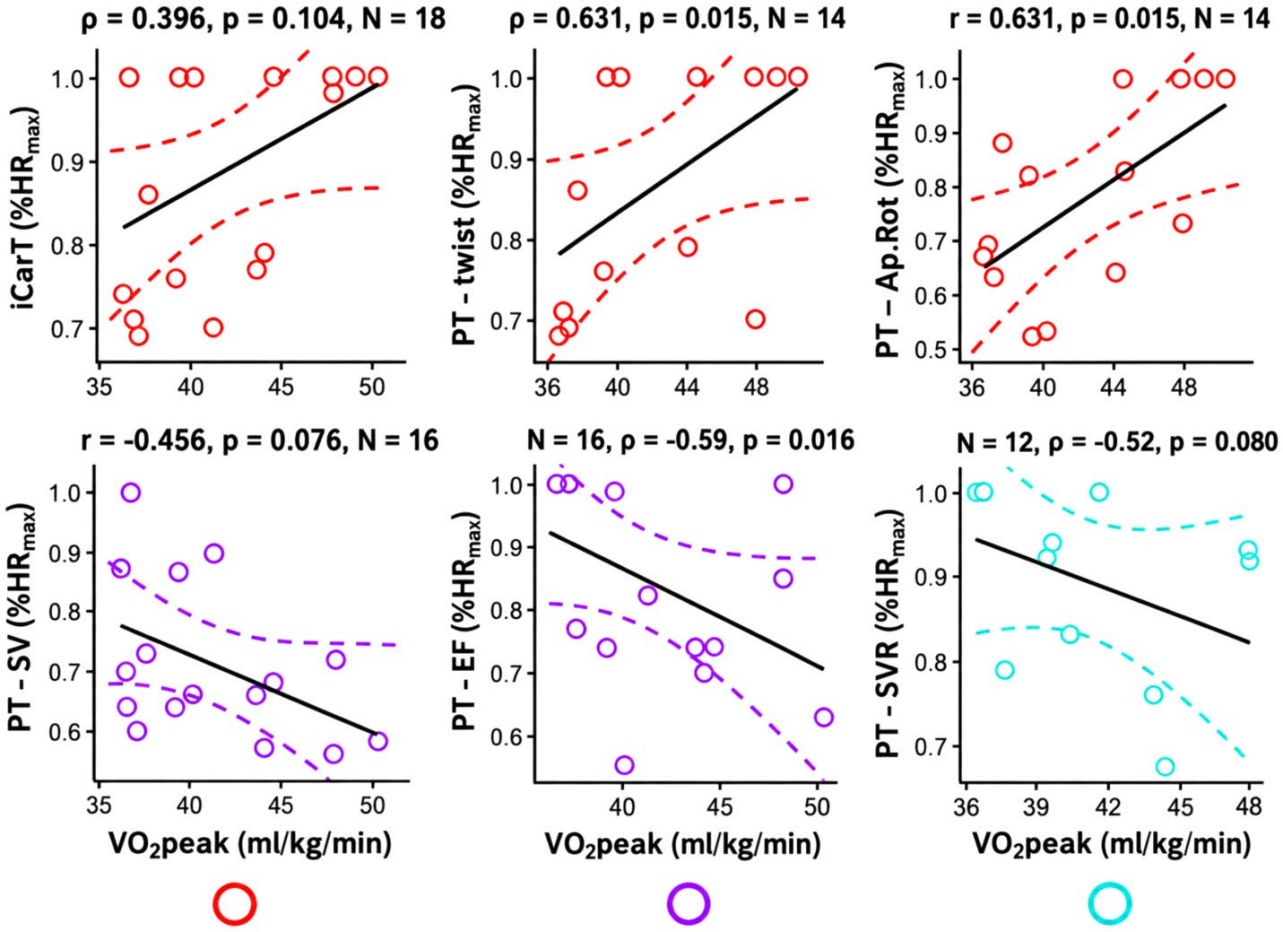
Associations between VO_2_peak and selected cardiovascular plateau timing. Scatterplots illustrating associations between VO_2_peak and plateau timing (PT, expressed as %HRmax) of selected cardiac deformation and haemodynamic parameters. Upper panels show associations with iCarT, twist, and apical rotation (red). Lower panels show associations with stroke volume (SV; purple), ejection fraction (EF; purple), and systemic vascular resistance (SVR; cyan). Solid lines represent linear regression fits and dashed lines indicate 95% confidence intervals. Pearson correlation coefficients (*r*) or Spearman rank correlation coefficients (*ρ*), along with *p*‐values and sample sizes, are reported in each panel.

Within left‐ventricular volumetric parameters, VO_2_peak showed moderate but non‐significant negative associations with PT‐SV (*r*  = −0.46, *p* = 0.076) and PT‐EDV (*ρ* = −0.28, *p* = 0.301), while a significant negative association was observed with PT‐EF (*ρ* = −0.59, *p* = 0.016). Regarding peripheral haemodynamics, VO_2_peak was negatively associated with PT‐SVR (ρ = −0.44, *p* = 0.148) and PT‐VC (*ρ* = −0.52, *p* = 0.080), without reaching statistical significance. No significant associations between VO_2_peak and plateau timing of other deformation parameters or iCarT (see Table 3) could be shown.

**TABLE 3:** Correlation of cardiovascular Plateau—Timing with VO_2_peak.

	Correlation with VO_2_peak (mL kg^−1^ min^−1^)	
	*r*/*ρ*	*p*
*Volumetrics, global cardiac function and LS (n = 16)*		
PT‐EDV	*ρ* = −0.28	0.301
PT‐ESV	*ρ* = −0.45	0.084
PT‐EF	*ρ* = −0.59	0.016*
PT‐SV	*r* = −0.46	0.076
PT‐Q	*ρ* = −0.35	0.180
*Myocardial deformation mechanics (n = 14) and iCarT (n = 18)*		
PT‐LS	*ρ* = −0.15	0.590
PT‐twist	*ρ* = 0.63	0.015*
PT‐Untwisting	*ρ* = 0.19	0.523
PT‐ACS	*ρ* = 0.01	0.970
PT‐Ap.Rot	*r* = 0.63	0.015*
iCarT	*ρ* = 0.40	0.104
*Blood pressure (n = 14)*		
PT‐SBP	*ρ* = −0.12	0.684
PT‐DBP	*ρ* = −0.09	0.756
PT‐MAP	*ρ* = 0.11	0.709
*Peripheral haemodynamics (n = 12)*		
PT‐VC	*ρ* = −0.52	0.080
PT‐SVR	*ρ*= −0.44	0.148

*Note:* PT denotes the relative heart rate at which the respective cardiovascular parameter reached its plateau or leveling‐off point during incremental exercise. Associations with VO_2_peak are reported as Pearson's correlation coefficient (*r*) or Spearman's rank correlation coefficient (*ρ*), depending on bivariate normality as assessed by the Shapiro–Wilk test. Sample size (*n*) varies across parameters due to differences in data availability.

* *p* < 0.05 indicates a statistically significant association.

### 3.3 Association Between VO_2_peak and Resting/Peak Cardiac Function

VO_2_peak was not significantly associated with SVrest, EDVrest, or HRrest (illustrated in Figure 3). VO_2_peak was significantly associated with SVpeak (*r* = 0.502, *p* = 0.048) and peak untwisting rate (*ρ* = −0.596, *p* = 0.028). No significant associations between VO_2_peak and peak deformation parameters, peripheral haemodynamics, or blood pressure (see Table 4) could be shown.

**FIGURE 3:**
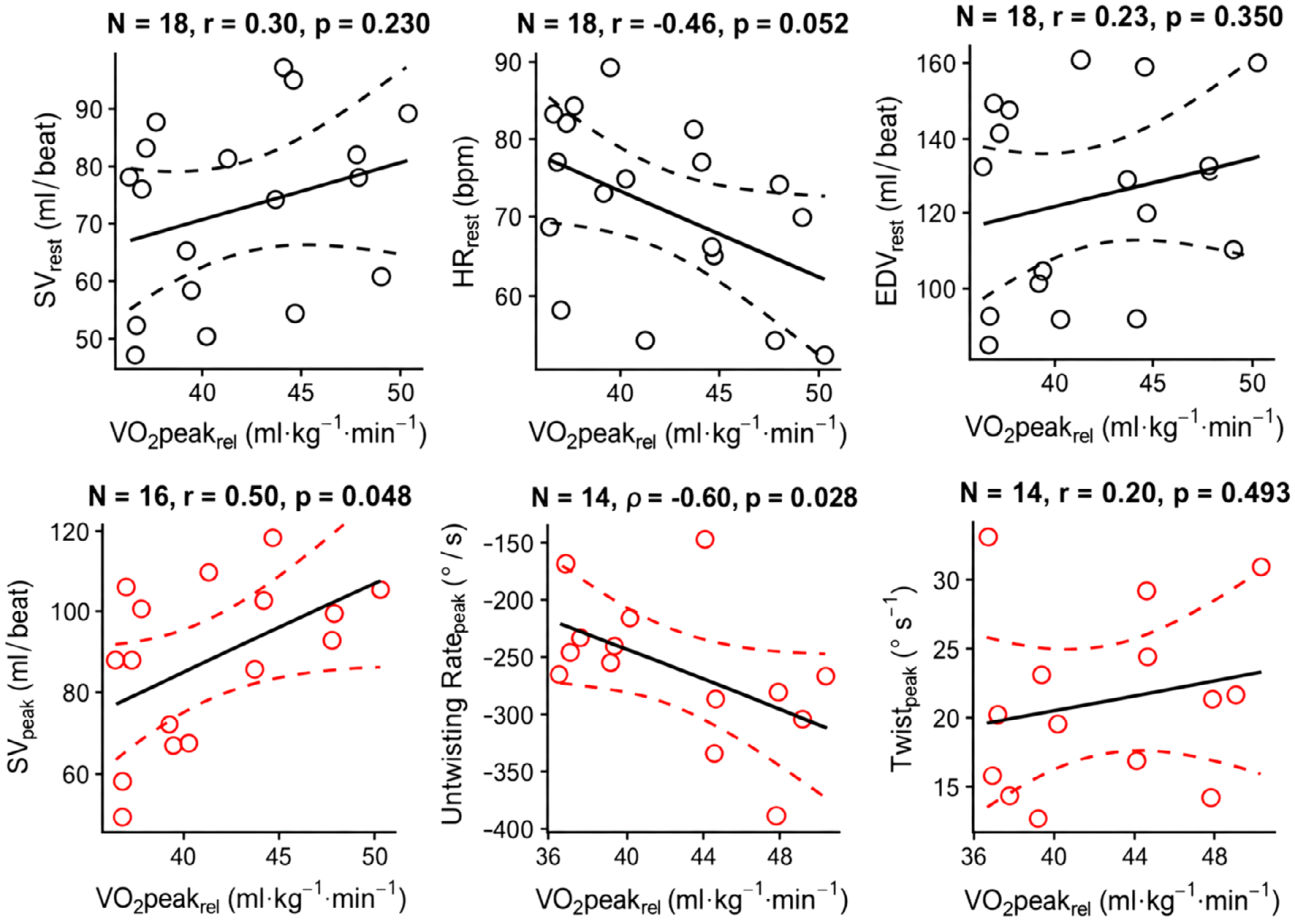
Associations between VO_2_peak and resting/peak cardiac function. Associations of VO_2_peak with selected cardiovascular variables at rest (black) and at peak exercise (red). Solid lines denote linear regression fits; dashed lines indicate 95% confidence intervals. Pearson correlation coefficients (*r*), *p*‐values, and sample sizes (*N*) are reported within each panel.

**TABLE 4:** Correlations Between VO_2_peak with Resting and Peak Cardiovascular Measures.

	Correlation with VO_2_peak (mL kg^−1^ min^−1^)	
	*r*/*ρ*	*p*
*Resting volumetric and global cardiac function (N = 18)*		
SV_rest_	*r* = 0.298	0.230
EDV_rest_	*r* = 0.234	0.350
HR_rest_	*r* = −0.464	0.052
*Peak volumetric and global cardiac function and LS (N = 16)*		
EDV_peak_	*r* = 0.429	0.097
ESV_peak_	*r* = 0.135	0.617
SV_peak_	*r* = 0.502	0.048*
Q_peak_	*r* = 0.17	0.541
EF_peak_	*r* = 0.213	0.446
LS_peak_	*r* = −0.383	0.143
*Peak LV rotational and circumferential deformation mechanics (N = 14)*		
Twist_peak_	*r* = 0.200	0.493
Untwisting_peak_	ρ = −0.596	0.028*
Ap.Rot_peak_	*r* = 0.376	0.185
ACS_peak_	*r* = −0.225	0.439
*Peak Blood pressure (N = 14)*		
SBP_peak_	*r* = 0.416	0.123
DBP_peak_	*r* = 0.258	0.373
MAP_peak_	*r* = 0.219	0.451
*Peak Peripheral haemodynamics (N = 12)*		
SVR_peak_	*r* = 0.279	0.379
VC_peak_	*r* = −0.110	0.735

*Note:* Correlations between aerobic capacity expressed (mL kg^−1^ min^−1^) and resting and peak cardiovascular parameters obtained during incremental exercise. Data are presented as Pearson's *r* or Spearman's *ρ* according to bivariate normality (Shapiro–Wilk test). Sample size (*n*) varies due to data availability.

* *p* < 0.05.

** *p* < 0.001.

### 3.4 Exploratory Clustering of Plateau‐Timing Response Patterns

Exploratory k‐means clustering based on *z*‐standardized domain‐level features of individual plateau timing was used to descriptively visualize the distribution of cardiovascular response patterns within the present sample. Two participants were excluded because of incomplete volumetric feature sets, leaving 16 participants for the analysis. Based on silhouette width, the data were represented by two exploratory groupings (*n* = 4 and *n* = 12; Figure 4).

**FIGURE 4:**
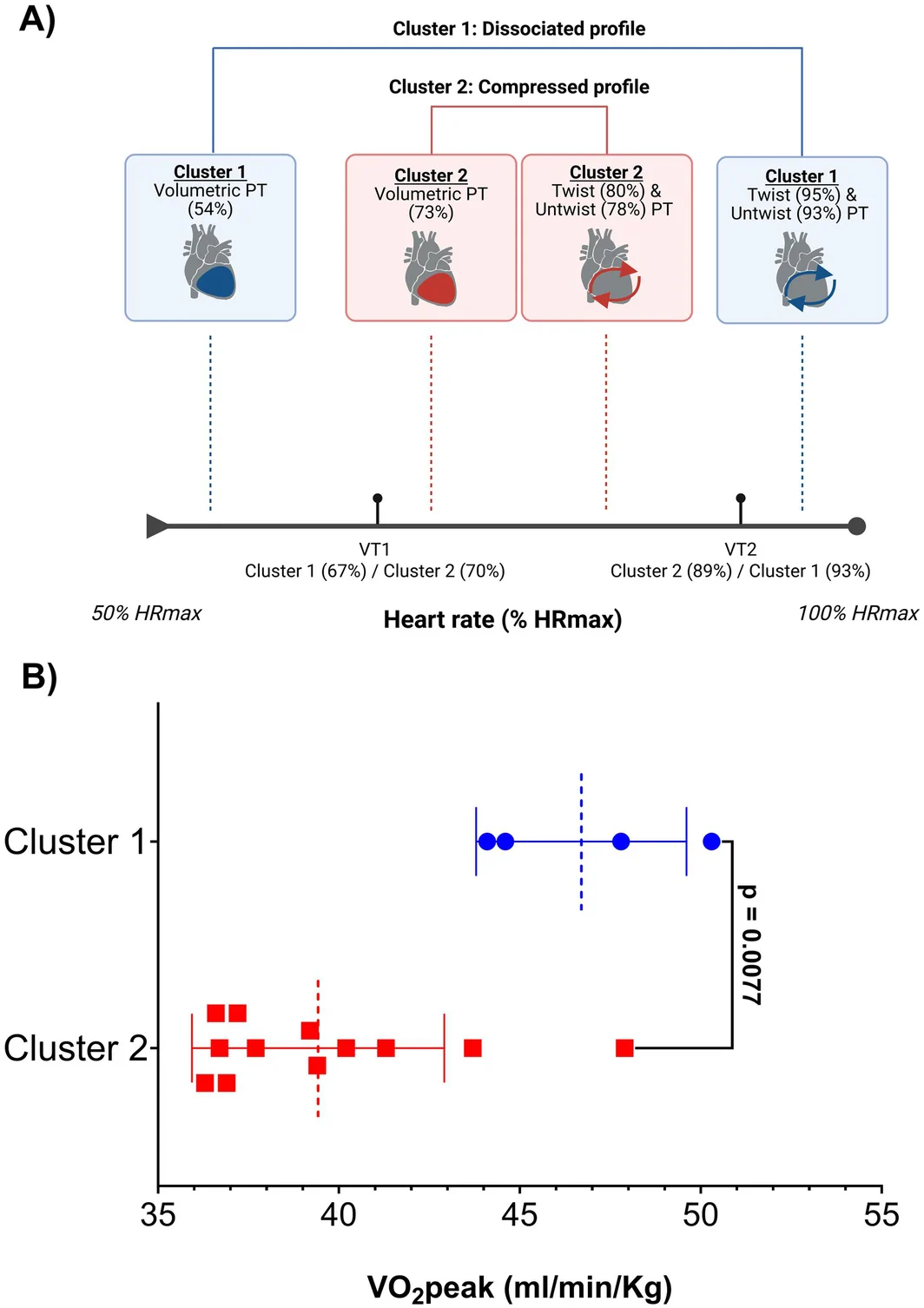
Schematic representation of exploratory cluster‐specific plateau‐timing profiles and association with VO_2_peak. (A) Schematic representation of the two exploratory plateau‐timing clusters. X‐axis represents %HRmax from 50% to 100%. Volumetric PT includes EDV, ESV, SV, and EF. Cluster 1 (higher VO2peak; dissociated profile) showed earlier volumetric plateau timing but later plateau timing of selected rotational mechanics, particularly twist and untwisting. In contrast, Cluster 2 (lower VO2peak; compressed profile) showed later volumetric plateau timing, while twist and untwisting plateaued earlier and in closer temporal proximity to the volumetric domain. The clustering analysis is intended as a descriptive visualization of interindividual variability. (B) Individual VO2peak values by cluster, shown with group summary. Cluster 1 exhibited a higher VO2peak than Cluster 2 (*p* = 0.0077).

The two profiles differed primarily in aerobic capacity, volumetric plateau timing and selected rotational parameters, whereas VT1 and VT2 were only moderately separated (see Figure 4). Cluster 1 exhibited a higher VO_2_peak than Cluster 2 (46.7 ± 2.9 vs. 39.4 ± 3.5 mL kg^−1^ min^−1^, *p* = 0.008), together with a slightly lower VT1 (66.5 ± 5.7 vs. 69.5% ± 4.3% HRmax) and a later VT2 (93.0 ± 5.0 vs. 89.4 ± 4.6% HRmax). Descriptively, Cluster 1 was characterized by very early volumetric plateau timings (e.g., ESV~36, EDV~46, SV~59, EF~77, overall volumetric PT~54 %HRmax), but markedly later deformation‐based limitation points, particularly in rotational mechanics (untwisting~93 and twist ~95 %HRmax). By contrast, Cluster 2 showed later volumetric plateau timing (ESV~58, EDV~71, SV~74, EF~90, overall volumetric PT~73% HRmax), a more compressed deformation profile (e.g., untwisting ~78 and twist ~80 %HRmax), and very late haemodynamic plateau behavior near VT2/peak (e.g., Q~92, DBP~93, VC~92% HRmax). Thus, the higher‐fitness profile was characterized not by a uniform rightward shift of all plateau timings, but by a greater temporal dissociation between earlier volumetric and later deformation‐based limitation points (see Table 5).

**TABLE 5:** Descriptive characteristics of the plateau‐timing profile derived from the final domain‐based k‐means clustering model (*n* = 16).

	Full study sample	Cluster 1	Cluster 2
*n*	18*	4	12
VO_2_peak	41.9 ± 4.8	46.7 ± 2.9	39.4 ± 3.5
VT1	68.39 ± 5.19	66.5 ± 5.7	69.5 ± 4.3
VT2	90.65 ± 4.65	93.0 ± 5.0	89.4 ± 4.6
Mean volumetric PT	68.3 ± 9.9	54.4 ± 5.0	73.0 ± 5.8
Mean deformation PT	77.0 ± 8.7	79.4 ± 7.4	75.4 ± 9.6
Mean haemodynamic PT	87.0 ± 7.9	83.9 ± 6.9	89.5 ± 5.5
iCarT	88.9 ± 13.1	94.8 ± 10.5	85.1 ± 13.5

*Note:* Values are mean ± SD. VO_2_peak is expressed as mL kg^−1^ min^−1^. VT1, VT2, PT, and iCarT are expressed as %HRmax. Domain‐level timing variables were first summarized at the individual level and then averaged across participants within each cluster. Mean volumetric PT comprised ESV, EDV, SV, and EF; mean deformation PT comprised basal rotation, basal circumferential strain, LS, apical circumferential strain, apical rotation, untwisting, and twist; and mean haemodynamic PT comprised systolic and diastolic blood pressure, MAP, VC, SVR, and Q. *Two participants were excluded from clustering because of incomplete volumetric data.

## 4 Discussion

The present study primarily investigated whether individual cardiovascular limitation points temporally align with ventilatory thresholds during incremental exercise. Within this framework, VT1 was associated selectively with plateau timing of ejection fraction and vascular conductance, whereas VT2 was associated predominantly with late myocardial deformation limits, particularly apical circumferential strain, apical rotation, and iCarT. No statistically supported VT2 associations were observed for volumetric or global haemodynamic plateau timing. In secondary exploratory analyses, aerobic fitness showed parameter‐specific associations with plateau timing and with resting and peak cardiovascular function. Specifically, higher VO_2_peak was associated with later plateau timing in rotational cardiac deformation but earlier PT‐EF and earlier PT‐SV without reaching statistical significance for association with PT‐SV. VO_2_peak also showed associations with SV_peak_ and peak diastolic function represented by untwisting rate but not peak systolic function. Importantly, the exploratory clustering provided a descriptive visualization of interindividual variability in cardiovascular plateau timing during incremental exercise. Across the two clusters, the overall sequence of earlier volumetric, intermediate deformation‐based, and later haemodynamic plateau timings was broadly preserved, while the relative spacing between these domains varied between individuals. Descriptively, the cluster with higher mean VO_2_peak showed earlier volumetric plateau timing alongside later rotational/deformation‐based plateau timing, whereas the cluster with lower mean VO_2_peak showed later volumetric and more closely spaced deformation timings, with haemodynamic plateaus occurring predominantly at higher exercise intensities. These exploratory patterns suggest that interindividual differences in aerobic fitness may coincide with differences in the relative temporal organization of cardiovascular responses. However, given the small and imbalanced cluster sizes, these patterns should be regarded as a descriptive representation of the present data distribution rather than evidence of distinct or reproducible physiological sub‐phenotypes.

### 4.1 Limits of Peripheral Haemodynamics and Cardiac Systolic Efficiency Around VT1

In the present study, VT1 was selectively associated with PT‐EF and PT‐VC, whereas no robust association was observed with PT‐SV or other cardiovascular plateau timings. These findings suggest that individuals in whom VT1 occurs at lower relative exercise intensities also tend to exhibit an earlier attenuation of peripheral vasodilatory responses and systolic emptying efficiency. Notably, the absence of an association between VT1 and PT‐SV indicates that VT1 does not simply reflect exhaustion of global cardiovascular reserve. Rather, the present findings raise the possibility that VT1 coincides with a physiological transition during which metabolic perturbations and cardiovascular regulatory processes become increasingly interconnected.

One physiologically plausible explanation involves the progressive activation of metabolically sensitive Group III and IV muscle afferents. Experimental studies have demonstrated that sympathetic nerve discharge during exercise is closely linked to intramuscular acid–base disturbances, implicating Group III and IV muscle afferents as important contributors to cardiovascular regulation during increasing exercise intensities [25, 26, 27, 28].

Increases in mean arterial pressure, calf vascular resistance, and muscle sympathetic nerve activity have been shown to correlate closely with intracellular H^+^ accumulation in exercising skeletal muscle [28, 29]. Furthermore, experimental attenuation of intramuscular H^+^ sensing markedly blunts sympathetic and pressor responses during exercise, supporting a causal role of metabolite‐sensitive muscle afferents in cardiovascular regulation [30]. Although the present study did not assess autonomic activity directly, these observations provide a biologically plausible framework through which the temporal association between VT1 and peripheral haemodynamic plateau timing may be interpreted. However, the present findings are observational and hypothesis‐generating in nature, precluding conclusions regarding causality or the directionality of the underlying physiological relationships. Future studies may attempt to experimentally shift some of the primary variables and assess whether this is also accompanied by a shift in other physiological responses.

Additional insight has been provided by studies employing heart rate variability analyses during incremental exercise. Several investigations have demonstrated that indices of vagal modulation, including high‐frequency power and non‐linear metrics such as DFA‐α1, exhibit characteristic breakpoints close to VT1, consistent with a withdrawal of parasympathetic influence and a progressive shift toward sympathetic predominance [17, 31]. Such autonomic adjustments could plausibly influence both vascular control and cardiac performance during exercise.

Within this framework, the observed association between VT1 and PT‐EF suggests that the first metabolic transition may be temporally associated with the attainment of a functional maximum in systolic emptying efficiency. A progressive attenuation of vasodilatory reserve could limit further reductions in afterload and thereby constrain additional increases in EF. Consistent with this interpretation, PT‐EF was strongly associated with both PT‐VC and PT‐SVR, suggesting a close temporal relationship between systolic efficiency and peripheral vascular function. In contrast, the absence of a robust association between VT1 and PT‐SV likely reflects the multifactorial regulation of stroke volume during exercise.

Collectively, these findings raise the possibility that VT1 may coincide with a coordinated shift in cardiovascular regulation characterized by changes in peripheral vascular responsiveness and systolic efficiency. However, the present observations are associative and do not establish causality. Future studies combining exercise echocardiography with direct measures of autonomic activity or targeted manipulation of preload, afterload, and vascular function are needed to determine whether the observed temporal alignment reflects a mechanistic interaction or parallel manifestations of broader physiological reorganization during exercise.

### 4.2 Association of VT2 With Apical Deformation Plateau Timing and iCarT

In contrast to VT1, VT2 was selectively associated with iCarT, PT‐Ap.Rot, and PT‐ACS, whereas no significant associations were observed for volumetric or global haemodynamic plateau timing. These findings suggest that the second ventilatory threshold is more strongly associated with myocardial deformation‐based responses than with volumetric or haemodynamic behavior. Inspection of the intensity‐referenced response patterns further indicated that ACS and apical rotation typically approached plateau behavior within the high‐intensity exercise domain, with earlier VT2 occurrence being associated with earlier attainment of these deformation‐based plateaus.

One physiologically plausible explanation is that the second metabolic transition, characterized by pronounced metabolic acidosis and the onset of respiratory compensation, and thus reflecting a substantially increased systemic and myocardial metabolic load [14, 15], coincides with a state in which maximal myocardial deformation is attained. As exercise intensity increases, heart rate rises and diastolic duration shortens, leaving progressively less time for ventricular filling [13, 32]. Because LV twist and especially subsequent diastolic untwisting contribute to rapid early filling during exercise, progressive shortening of diastole may reduce the scope for further deformational–mechanical augmentation at high intensities. In healthy individuals, LV untwisting is tightly temporally linked to early diastolic filling and appears to facilitate rapid filling when diastole shortens during exercise [33, 34]. Extending into higher intensities, LV twist mechanics increase initially but subsequently level off during incremental exercise, consistent with a progressive exhaustion of torsional reserve as diastolic filling time becomes increasingly constrained [12, 13].

Moreover, coronary perfusion of the left ventricle occurs predominantly during diastole. The progressive shortening of diastolic duration therefore narrows the temporal window available for myocardial perfusion on a beat‐to‐beat basis, despite the substantial exercise‐induced increase in coronary blood flow [35, 36, 37]. At the same time, myocardial oxygen demand rises markedly with increasing cardiac work. Heinonen highlighted that sustaining maximal cardiac performance requires a parallel augmentation of myocardial blood flow and that the capacity of the coronary circulation to meet the metabolic demands of the working myocardium may represent an important component of maximal cardiac output [38]. Although coronary blood flow was not assessed in the present study, these observations raise the possibility that the physiological reserve supporting further myocardial mechanical augmentation becomes progressively constrained at high exercise intensities.

In parallel, VT2 is characterized by profound metabolic and autonomic perturbations. Around this intensity domain, vagal modulation is largely withdrawn, heart rate variability approaches minimal values, and deviations from linear heart rate kinetics emerge in close proximity to VT2 [16, 17]. Simultaneously, increasing intracellular acidosis has been shown to impair contractile performance, reduce myofilament calcium sensitivity, and attenuate responsiveness to β‐adrenergic stimulation ([39, 40, 41]).

Taken together, these observations suggest that the physiological environment characterizing VT2 may coincide with conditions under which further augmentation of myocardial deformation becomes progressively constrained. While the underlying mechanisms cannot be determined from the present data, the combined influences of shortened diastolic filling time, increasing myocardial metabolic demand, autonomic reorganization, and intracellular acidosis provide a potential physiologically plausible framework for interpreting the observed temporal association between VT2 and deformation‐based plateau timing.

### 4.3 Associations Between Aerobic Fitness and Resting and Peak Left Ventricular Volumes and Cardiac Output

The present data indicate that higher aerobic fitness, expressed by VO_2_peak, was linked with favorable central cardiac characteristics at rest, although relationships did not reach statistical significance. Specifically, VO_2_peak showed positive associations with SV_rest_ and EDV_rest_, alongside a negative association with resting heart rate. The inverse relationship with HR was the most pronounced (*r* = −0.46, *p* = 0.052) and approached statistical significance, suggesting a physiologically meaningful trend toward improved cardiac efficiency with increasing aerobic fitness as already described by numerous studies [42, 43]. At peak exercise, VO_2_peak was positively associated with stroke volume SV_peak_, whereas no clear relationships were observed for EDV_peak_ or Q_peak_. A likely explanation for the predominantly non‐significant findings is the relatively small range of VO_2_peak within the present cohort. The limited variability in aerobic fitness reduces statistical power and restricts the ability to detect stronger associations. In more heterogeneous samples, encompassing a broader spectrum of fitness levels, clearer and statistically significant relationships between VO_2_peak and central cardiac parameters would be expected.

### 4.4 Associations Between Aerobic Capacity and Peak Systolic and Diastolic Function

Previous research has predominantly addressed chronic adaptations of left ventricular deformation under resting conditions and suggests that LV‐twist mechanics reflect training‐related remodeling rather than a uniform scaling with aerobic fitness. Cross‐sectional and longitudinal evidence indicates that highly trained individuals may have lower or similar resting LV twist compared with sedentary controls, with additional data pointing to phase‐ and modality‐specific adaptations across prolonged endurance training [20, 44, 45, 46]. Consistently, a meta‐analysis by Beaumont et al. reported no overall differences in resting LV twist between athletic and non‐athletic populations, but identified exercise‐type‐specific patterns, including reduced twist and apical rotation in elite endurance athletes [47]. Collectively, these findings indicate that resting LV twist reflects complex, long‐term adaptations to training load and modality rather than serving as a simple marker of aerobic capacity.

In contrast, evidence linking peak LV deformation during exercise to aerobic fitness remains very limited. To our knowledge, no previous study has comprehensively examined peak systolic deformation parameters—including LV twist, longitudinal strain, apical rotation, and circumferential strain—in relation to aerobic capacity during incremental exercise. In the present study, VO_2_peak was not significantly associated with peak systolic deformation across these parameters, although the direction of associations was generally consistent with higher peak deformation values in individuals with higher aerobic capacity. To more definitively determine whether higher aerobic capacity is associated with greater peak systolic deformation, future studies should compare well‐trained and untrained individuals across the full spectrum of fitness in age‐matched cohorts with sufficiently large sample sizes.

In contrast, a significant positive association was detected between VO_2_peak and peak untwisting rate, suggesting that aerobic fitness preferentially influences maximal diastolic function during exercise rather than systolic performance. Given the established role of untwisting in facilitating rapid early diastolic filling, this finding points to diastolic mechanics as a potential important link between aerobic fitness and peak cardiovascular performance [33]. When considered alongside previous work, these findings extend the emerging view that diastolic function contributes meaningfully to maximal aerobic performance. Studer Bruengger et al. reported positive associations between VO_2_max and post‐exercise indices of early diastolic function, including transmitral E velocity and tissue Doppler e' velocities, in a young and healthy cohort [48]. While those findings were based on conventional Doppler‐derived markers obtained post‐exercise, the present study extends this concept by capturing diastolic mechanics at peak exercise and linking aerobic capacity to a fundamental mechanical determinant of early filling. Thus, beyond established associations of aerobic capacity with SV_peak_, Q_peak_, and EDV_peak_, untwisting behavior may represent an additional functional marker of diastolic performance and aerobic fitness.

### 4.5 Modulation of Rotational Cardiac Contraction Limitations by Aerobic Capacity

An exploratory finding of the present study was that PT‐Ap.Rot and PT‐twist were positively associated with VO_2_peak. This parameter‐specific association was also reflected in the exploratory cluster visualization, where later rotational plateau timing in the higher‐VO_2_peak cluster was driven primarily by twist and untwisting rather than by a uniform delay of the entire deformation domain. These observations suggest that individuals with higher cardiorespiratory fitness tend to attain peak or plateau rotational mechanics at higher relative exercise intensities. Given that rotational deformation parameters generally plateau later than longitudinal and circumferential deformation during incremental exercise, a delayed plateau in LV twist and apical rotation may reflect a greater rotational mechanical reserve [12]. This interpretation is broadly consistent with previous observations in endurance‐trained individuals. Nottin et al. proposed that lower resting torsion may permit a greater torsional reserve during exercise, thereby facilitating a larger dynamic range of rotational adaptation [49]. In contrast, Cooke et al. revealed that a greater SV at rest and during submaximal exercise can be generated with a similar twist [44]. Similar observations have been made in pregnant and non‐pregnant women [50]. In addition, endurance‐trained individuals typically exhibit a greater heart rate reserve and enhanced exercise tolerance, which may allow rotational mechanics to be maintained across a broader range of exercise intensities before approaching plateau behavior [51]. Other physiological adaptations associated with endurance training, including enhanced diastolic function, altered β‐adrenergic responsiveness, and improved myocardial energetics, may also contribute to these observations [52, 53, 54].

Within this physiological context, it is noteworthy that rotational plateau timing occurred in close temporal proximity to VT2. Consistent with this observation, participants with higher VO_2_peak tended to exhibit both later rotational plateau timing and later attainment of VT2, although the association between VO_2_peak and relative exercise intensity at VT2 did not reach statistical significance. Taken together, these findings raise the possibility that cardiorespiratory fitness may influence the temporal organization of myocardial mechanical responses during exercise as illustrated in Figure 4. However, given the exploratory nature of these analyses and the relatively narrow fitness range of the present cohort, confirmation in larger and more diverse populations is required.

### 4.6 Inverse Association of Relative Aerobic Capacity With Volumetric Plateau Timing

Previous work from our group demonstrated that iCarT, derived from deformation‐based indices, does not coincide with the plateau of volumetric parameters [12]. Instead, LS, which plateaus earlier during incremental exercise than rotational and circumferential deformation at the LV apex, appears to more closely track stroke volume behavior, indicating that contractile and volumetric responses follow partially dissociated trajectories during incremental exercise (see Figure 4). Building on this concept, the present findings provide exploratory evidence that the temporal organization of central cardiovascular responses is not uniform and may be modulated in a parameter‐specific manner. In contrast to the later plateau timing of global and apical rotational mechanics observed with higher aerobic fitness, VO_2_peak showed inverse associations with the plateau timing of several volumetric and global haemodynamic parameters, including EF, EDV, SV, Q, and SVR. However, statistical significance was evident only for PT–EF. This pattern is noteworthy because greater aerobic capacity is usually associated with enhanced cardiovascular reserve [4] such that an earlier plateau of volumetric variables may appear counterintuitive at first glance.

A potential explanation may relate to differences in absolute cardiac size and operating range between individuals with higher and lower aerobic fitness. Endurance‐trained individuals typically exhibit larger EDV_peak_, SV_peak_, and Q_peak_, reflecting structural cardiac adaptations and expanded central blood volume. As a consequence, these individuals may reach their maximal volumetric filling and ejection capacity at lower relative exercise intensities, while still operating at substantially higher absolute volumes. In this scenario, an earlier plateau in SV or EDV does not indicate a functional limitation, but rather reflects that volumetric reserve is already fully recruited. Importantly, despite an earlier plateau in SV, Q can continue to increase through chronotropic mechanisms, given the preserved or even expanded heart rate reserve in trained individuals. In addition, the sustained increase in systolic rotational mechanics observed in individuals with higher aerobic capacity, including Ap.Rot and LV twist, may help maintain ventricular ejection across a broader range of workloads and attenuate the decline in SV at higher intensities. Within this framework, earlier plateauing of volumetric parameters may coexist with superior central delivery capacity, owing to larger absolute operating volumes, enhanced chronotropic flexibility, and a prolonged availability of systolic mechanical reserve in endurance‐trained individuals [42, 43].

Support for this line of reasoning can be drawn from exercise echocardiography data [55] which demonstrate that, at comparable heart rates in the low‐to‐moderate intensity domain, left ventricular twist and rotational mechanics are largely similar between fitness groups. However, with increases in heart rate, particularly approaching ~140 bpm, trained individuals show a markedly steeper increase in SV and diastolic filling rate, resulting in higher absolute values at this fixed heart rate, whereas corresponding responses in untrained individuals rise more modestly and tend to attenuate [55]. Importantly, neither plateau timings nor deformation responses were assessed beyond this heart rate range. In practical terms, the data of Fischer et al. suggest that endurance‐trained individuals exhibit a pronounced enhancement of diastolic filling and SV already during aerobic exercise in the low‐to‐submaximal intensity domain, compared with less trained individuals. This results in a steeper increase in volumetric filling and ejection at lower heart rates, enabling trained individuals to sustain higher external workloads at the same heart rate. When considered in relative terms, such an early and robust recruitment of volumetric function may contribute to an earlier attainment of near‐maximal volumetric capacity during incremental exercise, thereby predisposing volumetric parameters to plateau at lower relative intensities despite superior absolute performance. Moreover, the observation that rotational mechanics are comparable between trained and untrained individuals at heart rates around 140 bpm indicates that trained individuals do not rely on increased rotational deformation during the submaximal domain.

Collectively, the present findings suggest that aerobic capacity does not uniformly shift all cardiovascular peak or plateau responses toward higher relative exercise intensities. Instead, higher VO_2_peak was associated with earlier plateau timing of selected volumetric responses and later plateau timing of selected rotational mechanics (Figures 2 and 4). This pattern may indicate greater temporal separation between early volumetric recruitment and late rotational mechanical responses in individuals with higher aerobic capacity. However, this interpretation remains hypothesis‐generating, as only selected associations reached statistical significance, and the clustering analysis was of exploratory nature.

The exploratory cluster analysis is consistent with this parameter‐specific interpretation. Rather than identifying discrete physiological phenotypes, clustering provided a descriptive visualization of interindividual variability. The higher‐VO_2_peak cluster showed earlier volumetric plateau timing together with later selected rotational plateau timing, whereas the lower‐VO_2_peak cluster showed a more compressed pattern. Given the small sample size, unequal cluster sizes, and incomplete data across domains, these profiles should be considered hypothesis‐generating.

### 4.7 Limitations

The present findings should be interpreted in light of several limitations. Although the amount of work to generate the presented data is extensive, it has to be acknowledged that the sample size was modest and varied across outcome domains because not all measures were available in every participant. Longitudinal strain and volumetric measures were available in 16 participants, rotational and circumferential mechanics in 14 participants, and peripheral haemodynamics derived from beat‐to‐beat blood pressure in only 12 participants, which reduced statistical power, increased uncertainty of correlation estimates, and limited the robustness of domain‐specific conclusions. Particularly for the haemodynamic subset (*n* = 12), the limited sample size substantially reduced statistical power and increased the uncertainty of correlation estimates, such that moderate associations may have remained undetected and observed associations may be less precisely estimated. Findings from these smaller domain‐specific subsets should therefore be considered exploratory and require confirmation in larger independent samples. Furthermore, the exploratory k‐means analysis was based on a small sample (*n* = 16) with imbalanced cluster sizes (*n* = 4 vs. *n* = 12), increasing susceptibility to cluster instability and overfitting; the resulting patterns should therefore be interpreted descriptively rather than as distinct or reproducible physiological sub‐phenotypes.

The study design was observational and relied primarily on correlation analyses; therefore, causal inference is not possible and mechanistic interpretations concerning autonomic regulation, intracellular acid–base status, coronary perfusion, or local vascular control remain speculative because these processes were not measured directly. In addition, not all participants reached the predefined respiratory exchange ratio criterion during the second exercise test. Because this protocol was designed to optimize echocardiographic image acquisition throughout incremental exercise, some individuals may not have achieved the same degree of physiological exhaustion as during the dedicated cardiopulmonary exercise test. Consequently, the timing of peak or plateau responses in these participants may have been influenced by a slightly lower maximal exercise stimulus. Furthermore, plateau timing was derived from polynomial curve fitting of individual cardiovascular response patterns. Although this approach provides a standardized and reproducible framework for estimating participant‐specific peak or plateau timing across multiple physiological domains, the resulting plateau points should be interpreted as descriptive approximations rather than precise physiological breakpoints. In particular, the polynomial models may be sensitive to data density and measurement variability, especially at higher exercise intensities where fewer observations are available. The sensitivity analysis indicated that plateau estimates were generally stable at the group level, with only small average changes following removal of the final exercise‐stage observation, although larger deviations occurred in selected individuals and differed between physiological parameters. This individual variability highlights that polynomial curve fitting can be sensitive to data density and measurement variability, particularly at higher exercise intensities. At the same time, removal of the final observation inevitably reduces information from the highest‐intensity portion of the physiological response, which may impair the detection of late‐occurring plateaus and reduce inter‐individual resolution of iCarT. Accordingly, changes in the associations with ventilatory thresholds observed in the sensitivity analysis should be interpreted cautiously, as the sensitivity‐derived plateau estimates contain less information on late‐occurring functional limitations. In addition, ventilatory thresholds were determined using established criteria by experienced investigators; however, formal inter‐rater reliability was not assessed. Future studies should therefore formally evaluate the reproducibility and inter‐rater agreement of threshold determination procedures, particularly when ventilatory thresholds serve as primary variables in subsequent analyses. The study included only young, healthy, recreationally active individuals and used a supine exercise protocol with a 45° left‐lateral tilt to facilitate echocardiographic image acquisition, limiting generalisability to other populations and upright exercise conditions. Compared with upright cycling, supine exercise reduces gravitational blood pooling and is associated with greater cardiac filling and stroke volume, particularly at rest and during lower exercise intensities, whereas these postural differences tend to diminish at higher intensities [56, 57]. These posture‐dependent differences in cardiac loading conditions should therefore be considered when extrapolating the present findings to upright exercise. Finally, because multiple correlations were examined across several physiological domains, findings close to the conventional significance threshold should be interpreted cautiously and require confirmation in independent samples. In particular, the observed relationships between VT2 and ACS and between VT2 and iCarT should be considered indicative rather than conclusive, given their sensitivity to the robustness analyses.

## 5 Conclusion

The present study does not support the concept of a single uniform cardiovascular ceiling during incremental exercise. Instead, the findings indicate a parameter‐specific organization of peak cardiovascular responses across volumetric, haemodynamic, and myocardial deformation domains. Partly consistent with our hypotheses, VT1 was associated with the plateau timing of ejection fraction and vascular conductance, suggesting that the first cardiometabolic transition is linked to changes in systolic efficiency and peripheral vascular regulation. In contrast, the primary analyses suggest a potential relationship between VT2 and myocardial deformation plateau timing, particularly apical circumferential strain, apical rotation, and iCarT. However, the associations of VT2 and PT‐ACS as well as VT2 and iCarT require cautious interpretation in light of the sensitivity analysis. Aerobic fitness further influenced these response patterns. Higher VO_2_peak was associated with later plateau timing of rotational mechanics but earlier plateau timing of selected volumetric responses, suggesting that greater fitness reshapes the coordination of cardiovascular responses rather than uniformly delaying their peak timing. Collectively, these findings support the concept of an integrated cardiometabolic response to incremental exercise, in which cardiac mechanics, haemodynamics, and cardiometabolic thresholds are linked but exhibit domain‐specific and temporally staggered behavior. This work provides a foundation for future investigations into the mechanisms governing cardiometabolic integration during exercise.

## Author Contributions

**Fabian Spahiu:** conceptualization, investigation, writing – original draft, methodology, validation, visualization, writing – review and editing, project administration, data curation. **Moritz Lampkemeyer:** investigation, writing – original draft, visualization, writing – review and editing, software. **Michelle Ottlik:** investigation, data curation, visualization. **Max Hagemann:** investigation, methodology. **Lars C. Helbig:** investigation, methodology. **Eric J. Stöhr:** conceptualization, visualization, validation, supervision.

## Conflicts of Interest

The authors declare no conflicts of interest.

## Supporting information


**Table S1:** Participant‐level sensitivity analysis of plateau estimates following exclusion of the final available exercise‐stage observation.
**Table S2:** Parameter‐level sensitivity of plateau estimates following exclusion of the final available exercise‐stage observation.
**Table S3:** Associations between sensitivity‐derived plateau estimates and ventilatory thresholds.
**Figure S1**. Participant‐level data attrition across physiological outcome domains.

## Data Availability

The data that support the findings of this study are available on request from the corresponding author. The data are not publicly available due to privacy or ethical restrictions.
