# Supplementary material for: Peak Cardiac Mechanical and Haemodynamic Responses During Incremental Exercise Are Temporally Associated With Ventilatory Thresholds: Novel Insight Into the Coordination of Integrative Physiological Responses to Exercise
Source: Acta Physiol (Oxf). 2026 Nov 2;242(12):e70331. doi: 10.1111/apha.70331 (PMC13725837; doi:10.1111/apha.70331)
Supplement: Supplementary file 1 [file APHA-242-e70331-s001.docx]

**Supplemental Materials**

**Peak Cardiac Mechanical and Haemodynamic Responses During Incremental Exercise Are Temporally Associated with Ventilatory Thresholds**

**Supplementary Table S1**

**Participant-level sensitivity analysis of plateau estimates following exclusion of the final available exercise-stage observation**

**A. Myocardial deformation**

| **ID** | **PT - Twist** | | | **PT - Apical rotation** | | | **PT - ACS** | | | **PT - LS** | | | **iCarT** | | |
| --- | --- | --- | --- | --- | --- | --- | --- | --- | --- | --- | --- | --- | --- | --- | --- |
|  | **Original** | **Sensitivity** | **Δ** | **Original** | **Sensitivity** | **Δ** | **Original** | **Sensitivity** | **Δ** | **Original** | **Sensitivity** | **Δ** | **Original** | **Sensitivity** | **Δ** |
| **1** | 0,710 | 1,000 | 0,290 | 0,690 | 1,000 | 0,310 | 0,600 | 0,670 | 0,070 | 0,700 | 0,692 | -0,008 | 0,710 | 1,000 | 0,290 |
| **2** |  |  |  |  |  |  |  |  |  | 0,700 | 0,835 | 0,135 | 0,700 | 0,835 | 0,135 |
| **3** | 1,000 | n.b. | n.b. | 0,530 | n.b. | n.b. | 0,570 | n.b. | n.b. | 0,560 | 0,545 | -0,015 | 1,000 | 0,545 | -0,455 |
| **4** | 0,790 | 0,768 | -0,022 | 0,640 | 0,616 | -0,024 | 0,550 | 0,571 | 0,021 | 0,670 | 0,726 | 0,056 | 0,790 | 0,768 | -0,022 |
| **5** | 0,760 | 0,639 | -0,121 | 0,820 | 0,778 | -0,042 | 1,000 | 0,755 | -0,245 | 0,470 | 1,000 | 0,530 | 0,760 | 1,000 | 0,240 |
| **6** |  |  |  |  |  |  |  |  |  | 0,740 | 1,000 | 0,260 | 0,740 | 1,000 | 0,260 |
| **7** | 0,690 | 0,599 | -0,091 | 0,630 | 0,616 | -0,014 | 0,580 | 1,000 | 0,420 | 0,610 | 0,610 | 0,000 | 0,690 | 1,000 | 0,310 |
| **8** |  |  |  |  |  |  |  |  |  | 1,000 | 1,000 | 0,000 | 1,000 | 1,000 | 0,000 |
| **9** |  |  |  |  |  |  |  |  |  | 0,770 | 1,000 | 0,230 | 0,770 | 1,000 | 0,230 |
| **11** | 0,700 | 0,744 | 0,044 | 0,730 | 0,739 | 0,009 | 0,700 | 0,764 | 0,064 | 0,980 | 1,000 | 0,020 | 0,980 | 1,000 | 0,020 |
| **12** | 1,000 | 1,000 | 0,000 | 0,520 | 0,487 | -0,033 | 1,000 | 1,000 | 0,000 | 1,000 | 1,000 | 0,000 | 1,000 | 1,000 | 0,000 |
| **14** | 1,000 | 1,000 | 0,000 | 0,830 | 1,000 | 0,170 | 0,790 | 0,813 | 0,023 |  |  |  | 1,000 | 1,000 | 0,000 |
| **15** | 1,000 | 0,931 | -0,069 | 1,000 | 1,000 | 0,000 | 1,000 | 1,000 | 0,000 | 0,710 | 0,701 | -0,009 | 1,000 | 1,000 | 0,000 |
| **17** | 1,000 | 1,000 | 0,000 | 1,000 | 1,000 | 0,000 | 0,630 | 1,000 | 0,370 |  |  |  | 1,000 | 1,000 | 0,000 |
| **18** | **0,860** | **0,784** | **-0,076** | **0,880** | **0,692** | **-0,188** | **0,730** | **0,675** | **-0,055** | **0,690** | **1,000** | **0,310** | **0,860** | **1,000** | **0,140** |
| **19** | **0,680** | **1,000** | **0,320** | **0,670** | **0,696** | **0,026** | **1,000** | **1,000** | **0,000** | **0,660** | **0,644** | **-0,016** | **1,000** | **1,000** | **0,000** |
| **20** | **1,000** | **0,587** | **-0,413** | **1,000** | **1,000** | **0,000** | **0,780** | **0,527** | **-0,253** | **0,600** | **0,581** | **-0,019** | **1,000** | **0,587** | **-0,413** |
| **21** | **1,000** | **0,777** | **-0,223** | **1,000** | **1,000** | **0,000** | **1,000** | **1,000** | **0,000** | **0,550** | **0,562** | **0,012** | **1,000** | **1,000** | **0,000** |

**B. Volumetric and cardiac functional parameters**

| **ID** | **PT - EF** | | | **PT - SV** | | | **PT - Q** | | |
| --- | --- | --- | --- | --- | --- | --- | --- | --- | --- |
|  | **Original** | **Sensitivity** | **Δ** | **Original** | **Sensitivity** | **Δ** | **Original** | **Sensitivity** | **Δ** |
| 1 | 1.000 | 0.848 | -0.152 | 1.000 | 1.000 | 0.000 | 1.000 | 0.893 | -0.107 |
| 2 | 0.820 | 1.000 | +0.180 | 0.890 | 1.000 | +0.110 | 1.000 | 1.000 | 0.000 |
| 3 | 0.550 | 0.595 | +0.045 | 0.650 | 0.646 | -0.004 | 0.800 | 0.804 | +0.004 |
| 4 | 0.700 | 0.774 | +0.074 | 0.560 | 0.592 | +0.032 | 0.750 | 0.774 | +0.024 |
| 5 | 0.740 | 0.724 | -0.016 | 0.630 | 0.567 | -0.063 | 1.000 | 1.000 | 0.000 |
| 6 | 1.000 | 1.000 | 0.000 | 0.870 | 1.000 | +0.130 | 0.900 | 1.000 | +0.100 |
| 7 | 1.000 | 1.000 | 0.000 | 0.590 | 0.740 | +0.150 | 0.800 | 0.832 | +0.032 |
| 8 | 1.000 | 0.540 | -0.460 | 0.630 | 0.629 | -0.001 | 0.850 | 1.000 | +0.150 |
| 9 | 0.740 | 1.000 | +0.260 | 0.650 | 0.655 | +0.005 | 0.780 | 0.788 | +0.008 |
| 11 | 0.850 | 0.638 | -0.212 | 0.710 | 0.623 | -0.087 | 0.950 | 1.000 | +0.050 |
| 12 | 0.990 | 0.596 | -0.394 | 0.860 | 0.565 | -0.295 | 0.900 | 0.584 | -0.316 |
| 15 | 0.740 | 0.815 | +0.075 | 0.670 | 0.732 | +0.062 | 0.810 | 0.851 | +0.041 |
| 18 | 0.770 | 0.782 | +0.012 | 0.720 | 0.739 | +0.019 | 0.900 | 0.760 | -0.140 |
| 19 | 1.000 | 0.623 | -0.377 | 0.690 | 0.525 | -0.165 | 1.000 | 1.000 | 0.000 |
| 20 | 0.630 | 1.000 | +0.370 | 0.570 | 1.000 | +0.430 | 0.890 | 1.000 | +0.110 |
| 21 | 1.000 | 0.536 | -0.464 | 0.550 | 0.557 | +0.007 | 0.930 | 0.591 | -0.339 |

**C. Peripheral haemodynamics**

| **ID** | **PT - VC** | | | **PT - SVR** | | |
| --- | --- | --- | --- | --- | --- | --- |
|  | **Original** | **Sensitivity** | **Δ** | **Original** | **Sensitivity** | **Δ** |
| 2 | 1.000 | 1.000 | 0.000 | 1.000 | 1.000 | 0.000 |
| 3 | 0.830 | 0.710 | -0.120 | 0.830 | 0.715 | -0.115 |
| 4 | 0.670 | 0.658 | -0.012 | 0.680 | 0.671 | -0.009 |
| 5 | 0.920 | 0.940 | +0.020 | 0.870 | 0.843 | -0.027 |
| 6 | 1.000 | 1.000 | 0.000 | 1.000 | 1.000 | 0.000 |
| 8 | 1.000 | 1.000 | 0.000 | 1.000 | 1.000 | 0.000 |
| 9 | 0.760 | 1.000 | +0.240 | 0.760 | 1.000 | +0.240 |
| 11 | 0.920 | 0.795 | -0.125 | 0.900 | 0.795 | -0.105 |
| 12 | 0.940 | 1.000 | +0.060 | 0.880 | 1.000 | +0.120 |
| 18 | 0.790 | 1.000 | +0.210 | 0.800 | 1.000 | +0.200 |
| 19 | 1.000 | 0.804 | -0.196 | 1.000 | 1.000 | 0.000 |
| 21 | 0.930 | 0.586 | -0.344 | 0.870 | 0.615 | -0.255 |

Note. Plateau timing is expressed relative to maximum heart rate (%HRmax), where 1.00 corresponds to 100% HRmax. Sensitivity estimates were obtained by repeating the original curve-fitting procedure after exclusion of the final available exercise stage for each participant and parameter. Δ = sensitivity estimate − original estimate; positive values indicate a shift toward a later plateau and negative values a shift toward an earlier plateau. PT; plateau timing, ACS, apical circumferential strain; EF, ejection fraction; LS, longitudinal strain; Q, cardiac output; SV, stroke volume; SVR, systemic vascular resistance; VC, vascular conductance.

**Supplementary Table S2**

**Parameter-level sensitivity of plateau estimates following exclusion of the final available exercise-stage observation**

| **Parameter** | **n** | **Mean original plateau** | **Mean sensitivity plateau** | **Mean Δ** | **Mean \|Δ\|** | **Median \|Δ\| (Q1–Q3)** | **Range Δ** |
| --- | --- | --- | --- | --- | --- | --- | --- |
| **Myocardial deformation** | | | | | | | |
| PT - Twist | 13 | 0.861 | 0.833 | -0.028 | 0.128 | 0.076 (0.022–0.223) | -0.413 to +0.320 |
| PT - Apical rotation | 13 | 0.801 | 0.817 | +0.016 | 0.063 | 0.024 (0.000–0.042) | -0.188 to +0.310 |
| PT - ACS | 13 | 0.797 | 0.829 | +0.032 | 0.117 | 0.055 (0.000–0.245) | -0.253 to +0.420 |
| PT - LS | 16 | 0.713 | 0.806 | +0.093 | 0.101 | 0.018 (0.009–0.159) | -0.019 to +0.530 |
| **Volumetric and cardiac functional parameters** | | | | | | | |
| PT - EF | 16 | 0.846 | 0.779 | -0.066 | 0.193 | 0.166 (0.038–0.372) | -0.464 to +0.370 |
| PT - SV | 16 | 0.703 | 0.723 | +0.021 | 0.098 | 0.062 (0.006–0.135) | -0.295 to +0.430 |
| PT - Q | 16 | 0.891 | 0.867 | -0.024 | 0.089 | 0.045 (0.007–0.117) | -0.339 to +0.150 |
| **Peripheral haemodynamics** | | | | | | | |
| PT - VC | 12 | 0.897 | 0.874 | -0.022 | 0.111 | 0.090 (0.009–0.200) | -0.344 to +0.240 |
| PT - SVR | 12 | 0.883 | 0.886 | +0.004 | 0.089 | 0.066 (0.000–0.140) | -0.255 to +0.240 |

Note. Plateau timing is expressed relative to maximum heart rate (%HRmax), where 1.00 corresponds to 100% HRmax. The mean original plateau and mean sensitivity plateau represent the arithmetic mean of participant-level plateau estimates before and after exclusion of the final available exercise stage, respectively. Δ was calculated as sensitivity estimate − original estimate. Mean Δ therefore quantifies the average directional shift, whereas mean |Δ| quantifies the average absolute individual change irrespective of direction. Median |Δ| is presented with the first and third quartiles (Q1–Q3), and Range Δ represents the minimum and maximum signed individual changes. For PT - Twist, PT - apical rotation, and PT - ACS, participant ID 3 was excluded from paired summary statistics because no plateau could be determined after exclusion of the final observation stage. PT; plateau timing, ACS, apical circumferential strain; EF, ejection fraction; LS, longitudinal strain; Q, cardiac output; SV, stroke volume; SVR, systemic vascular resistance; VC, vascular conductance.

**Supplementary Table S3**

**Associations between sensitivity-derived plateau estimates and ventilatory thresholds**

| **Parameter** | **n** | **VT1: Spearman ρ** | **p** | **VT2: Spearman ρ** | **p** |
| --- | --- | --- | --- | --- | --- |
| iCarT | 17 | 0.331 | 0.194 | 0.131 | 0.617 |
| PT - Twist | 13 | 0.037 | 0.904 | 0.011 | 0.971 |
| PT - Apical rotation | 13 | -0.301 | 0.318 | 0.304 | 0.313 |
| PT - ACS | 13 | 0.747 | 0.003 | 0.309 | 0.304 |
| PT - LS | 16 | -0.034 | 0.901 | 0.003 | 0.991 |
| PT - EF | 16 | -0.409 | 0.116 | -0.331 | 0.210 |
| PT - SV | 16 | -0.560 | 0.024 | -0.490 | 0.054 |
| PT - Q | 16 | -0.022 | 0.937 | -0.197 | 0.466 |
| PT - VC | 12 | -0.110 | 0.732 | 0.007 | 0.982 |
| PT - SVR | 12 | 0.074 | 0.818 | 0.023 | 0.942 |

Note. Values are Spearman rank correlation coefficients (ρ). Plateau estimates were obtained after exclusion of the final available exercise-stage observation. Bold values indicate p < 0.05. iCarT was defined as the latest plateau among the available myocardial deformation parameters (LS, twist, and ACS); when only one deformation parameter was available, that parameter was used. For ID_003, iCarT could not be determined after exclusion of the final observation. ACS, apical circumferential strain; EF, ejection fraction; iCarT, individual cardiac contraction threshold; LS, longitudinal strain; Q, cardiac output; SV, stroke volume; SVR, systemic vascular resistance; VC, vascular conductance; VT1, first ventilatory threshold; VT2, second ventilatory threshold.

**Supplementary Figure F1**

**Participant-level data attrition across physiological outcome domains**

Participant-level data availability and reasons for missing measurements are shown for apical four-chamber-derived, parasternal short-axis-derived, and peripheral haemodynamic analyses. Percentages refer to the total sample (N = 18). ACS, apical circumferential strain; EF, ejection fraction; LS, longitudinal strain; Q, cardiac output; SV, stroke volume; SVR, systemic vascular resistance; VC, vascular conductance.
